# Assessment of Age and Sex Difference in Cardiopulmonary Function of Children and Adolescents with Ventricular Septal Defect

**DOI:** 10.3390/jcdd12060204

**Published:** 2025-05-28

**Authors:** Chao-Ruei Lin, Ya-Fen Liu, I-Ching Huang, Yi-Jen Chen, Chia-Hsin Chen, Ko-Long Lin

**Affiliations:** 1 Department of Physical Medicine and Rehabilitation, Kaohsiung Municipal Siaogang Hospital, Kaohsiung Medical University, Kaohsiung 80756, Taiwan; 1080288@kmuh.org.tw (C.-R.L.); 900193@kmuh.org.tw (Y.-F.L.); 100311038@gms.tcu.edu.tw (I.-C.H.); 1100424@kmuh.org.tw (Y.-J.C.); 2 Department of Physical Medicine and Rehabilitation, Kaohsiung Medical University Gangshan Hospital, Kaohsiung Medical University, Kaohsiung 80756, Taiwan; 3 Department of Physical Medicine and Rehabilitation, Kaohsiung Medical University Hospital, Kaohsiung Medical University, Kaohsiung 80756, Taiwan; chchen@kmu.edu.tw; 4 Department of Physical Medicine and Rehabilitation, School of Medicine, College of Medicine, Kaohsiung Medical University, Kaohsiung 80756, Taiwan; 5 Neurotechnology and Assistive Technology Center, Kaohsiung Medical University, Kaohsiung 80756, Taiwan

**Keywords:** cardiopulmonary function, exercise testing, pediatric, ventricular septal defect, body mass index, sex

## Abstract

In the past, an isolated ventricular septal defect (VSD) was categorized as a simple lesion with assumed negligible long-term risks when treated correctly in childhood; however, evidence has suggested that patients with isolated congenital VSDs carry a substantial burden of cardiovascular morbidity throughout life. This study aimed to investigate the cardiopulmonary function (CPF) in isolated VSD patients of a young age and the relationship of CPF with age, sex and body mass index (BMI). This retrospective cohort study was conducted at a tertiary medical center in southern Taiwan. We recruited children and adolescents (aged 5 to 18 years) with isolated VSDs who underwent the symptom-limited treadmill exercise test. Data from 289 children and adolescents (157 males, 132 females) were included in the analysis. The participants were stratified into three groups according to age. The male patients had a significantly higher oxygen uptake at the anaerobic threshold (AT VO_2_) and a higher peak oxygen uptake (peak VO_2_) (all *p* < 0.05). Normalized AT VO_2_ (mL/kg/min) and normalized peak VO_2_ (mL/kg/min) were significantly negatively associated with BMI. In conclusion, male patients with isolated VSDs had similar or better CPF compared with female patients. BMI had a negative association with CPF in young VSD patients.

## 1. Introduction

A ventricular septal defect (VSD) is the most common type of congenital heart disease (CHD). The global prevalence of VSD is 30.71 per 10,000 births, and VSD contributes 35.56% to the total burden of CHD [1]. The prevalence of VSDs in Taiwan is 29.47 per 10,000 births [2]. VSDs can occur in isolation or as an integral component of complex lesions and can be further categorized by their location and the structures along their borders [3]. A septal defect might spontaneously close or require interventional closure.

In the past, an isolated VSD was categorized as a simple lesion with assumed negligible long-term risks when treated correctly in childhood. However, the evidence suggests that patients with isolated congenital VSDs carry a substantial burden of cardiovascular morbidity throughout life, including arrhythmias, infectious endocarditis, heart failure and pulmonary arterial hypertension [4]. Patients with isolated VSDs also have a lower survival compared with that of the general population [5].

An increasing number of studies has shown that adults with isolated VSDs who have undergone surgical closure in early childhood have a reduced pulmonary function, impaired right ventricular function and a lower peak heart rate and exercise capacity [6,7,8,9]. Even patients with isolated VSDs that are considered hemodynamically insignificant have demonstrated significantly impaired minute ventilation (VE), peak oxygen uptake (peak VO_2_) and biventricular contractility and abnormal right ventricular function [8,10]. Patients with isolated VSDs have demonstrated a worse exercise capacity than that of their healthy peers. This difference increases with advancing age and increases mostly in patients with operated isolated VSDs [11].

Cardiopulmonary fitness (CPF) is defined as the overall capacity of the cardiovascular and respiratory systems to carry out prolonged strenuous exercise and is one of the most important components of physical fitness related to lower morbidity and mortality in healthy adults [12]. Cardiopulmonary exercise testing (CPET) is considered the gold standard for measuring CPF, providing valuable diagnostic and prognostic information and guidance for safe participation in exercise [12,13]. Several parameters have been widely used as indicators of CPF, including VO_2_ at the anaerobic threshold (AT) and peak VO_2_.

Sex differences in prognosis have been reported in adults with congenital heart disease. In a previous study, females were at higher risk of pulmonary hypertension and at lower risk of poor aortic outcomes, endocarditis and implantable cardioverter–defibrillator complications [14]. Although the mechanism of the sex difference remains uncertain, exercise capacity could be a good predictor of late complications and major adverse cardiovascular events in CHD [15,16]. No study has directly discussed the sex difference in CPF in patients with CHD; however, a trend of males having better CPF than females is noticed within the available data [15,17]. In children and adolescents with CHD, males have better CPF compared with females, and the sex difference is less than that in healthy controls [15]. Obesity also appears to be an important issue for patients with CHD, and up to 25% of children with congenital heart disease are obese or overweight [18]. Such a condition in these patients might increase their cardiovascular risk and impair their exercise capacity [19].

Although VSDs are the most common form of CHD, the existing data on the exercise performance of children with isolated VSDs remain scarce and inconsistent [20,21,22,23]. This study therefore aimed to investigate the cardiopulmonary function of isolated VSD patients of a young age and the relationship of cardiopulmonary function with age, sex and body mass index (BMI).

## 2. Materials and Methods

### 2.1. The Study Design

This retrospective cohort study was conducted at a tertiary medical center in southern Taiwan and was approved by the Institutional Review Board (No. VGHKS15-CT7-05). We recruited children and adolescents (aged 5 to 18 years) with isolated VSDs who underwent regular follow-ups at a clinic between September 2015 and August 2022. The inclusion criteria were children with isolated VSDs who had undergone the symptom-limited treadmill exercise test under the ramped Bruce protocol. The exclusion criteria were (a) incomplete CPET and (b) recent hospitalization due to heart disease or other major diseases. Thei basic demographics was recorded, including age, sex, weight, height and BMI.

### 2.2. The Cardiopulmonary Exercise Testing (CPET)

Informed consent was acquired prior to the tests. Before the treadmill exercise testing, each patient was familiarized with the procedures and equipment, and they underwent the exercise testing according to the ramped Bruce protocol using the MetaLyzer 3B (Cortex Biophysik GmbH Co., Leipzig, Germany) system. Throughout the test, the participant’s blood pressure, heart rate (HR), respiratory exchange ratio (RER), minute ventilation (VE), oxygen consumption (VO_2_) and carbon dioxide production (VCO_2_) were recorded and monitored. The test was terminated when the patients demonstrated unbearable symptoms. All CPET trials were performed under the supervision of an experienced physiatrist.

The main CPF indicators included VO_2_ at the anaerobic threshold (AT) and peak VO_2_. We divided AT VO_2_ and peak VO_2_ by body mass and expressed this as the normalized AT VO_2_ (mL/kg/min) and normalized peak VO_2_ (mL/kg/min) for short. The anaerobic threshold (AT) was determined using the VE/VO_2_ and VE/VCO_2_ methods [24]. Physiological criteria for the completion of a valid peak VO_2_ test included two of the following three criteria: (1) a peak HR within 5% of the age-predicted maximum, (2) a RER ≥ 1.0, and (3) volitional fatigue [13,25].

### 2.3. The Statistical Analysis

The statistical analyses were performed using SPSS Statistics version 20 (IBM Corp., Armonk, New York, NY, USA). Continuous data was expressed as means ± standard deviation, and categorical variables were presented as absolute numbers and percentages. Normality and homoscedasticity were checked prior to each analysis. The correlation between BMI and aerobic fitness (AT VO_2_, peak VO_2_ and peak PD) in all subjects was examined using a Pearson’s correlation analysis for normally distributed variables and a Spearman’s correlation analysis for non-normally distributed variables. A *p*-value ≤ 0.05 was considered significant.

## 3. Results

A total of 303 patients were recruited initially, of whom 9 (2.7%) were excluded due to incomplete data records. Five (1.5%) patients who failed to meet the criteria for maximal effort were also excluded. Finally, data from 289 children and adolescents (157 males, 132 females) were included in the analysis. The participants were stratified into three groups according to age: group 1 (aged 5–9) included 44 males and 35 females; group 2 (aged 10–13) included 44 males and 50 females; and group 3 (aged 14–18) included 69 males and 47 females. The demographic results are presented in Table 1. No differences in body weight, body height or BMI were noted between the males and females in groups 1 and 2. The boys in group 3 had significantly higher body weights, body heights and BMIs compared with these values for the girls in this group.

Table 2 presents the differences in CPF between sexes in the three age groups. Boys had significantly higher AT VO_2_ values and peak VO_2_ values than girls in the two older groups (all *p* < 0.05). No significant differences in AT VO_2_ and peak VO_2_ between the males and females in group 1 were found; overall, the boys had significantly higher AT VO_2_ and higher peak VO_2_ values than the girls (all *p* < 0.05).

The correlation between BMI and performance in the exercise test is presented in Table 3. Absolute AT VO_2_ (mL/min) and peak VO_2_ (mL/min) were positively associated with BMI. The correlation coefficient ranged from 0.164 to 0.644 for AT VO_2_ (mL/min) and from 0.151 to 0.664 for peak VO_2_ (mL/min). The normalized AT VO_2_ and normalized peak VO_2_ were significantly negatively associated with BMI. The correlation coefficient ranged from −0.580 to −0.100 for normalized AT VO_2_ and from −0.599 to −0.329 for normalized peak VO_2_, respectively.

## 4. Discussion

This was a retrospective study that recruited more than 250 patients with isolated VSDs aged younger than 18. The results revealed that the male patients with isolated VSDs had a similar or better CPF compared with the female patients. In addition, BMI had negative association with CPF in the children with isolated VSDs.

According to the growth charts for Taiwanese children and adolescents, the body weights and body heights of our patients were around the 50th percentile, and nearly half of our patients were within the normal BMI group [26]. In our study, significant differences in body weight, body height and BMI between males and females were noticed in the isolated VSD patients in group 3 (ages 14–18). A similar pattern was also noticed in a previous study of healthy children and adolescents [27]. In normal children within the same age range, this phenomenon could also be seen in the growth charts, in which the 50th percentile for body weight and body height for girls fell within the 25th to 50th percentile for boys aged 5 to 14 and within the 3rd to 25th percentile for boys aged 15 to 17, indicating different ranges of growth spurts between sexes [26].

In our study, peak VO_2_ increased with age in both sexes, but males had significantly higher AT VO_2_ and higher peak VO_2_ values than those of females in groups 2 and 3; however, no significant differences in AT VO_2_ and peak VO_2_ were found between males and females in group 1 (age 5–9). The difference in the peak VO_2_ between males and females also increased with age [27,28]. In healthy children and adolescents from Taiwan, males had an 8.1% to 25.2% higher normalized peak VO_2_ compared to that for females in one study [27]. In other studies, males had a 10.6% to 32.4% higher normalized peak VO_2_ compared to that in females [29,30,31]. In our study, males only had a 1.9% to 17.7% higher normalized peak VO_2_ compared to that in females. The differences in CPF between males and females could be partially explained by the differences in their fat-free mass and hemoglobin concentrations [28,32]; furthermore, females usually have more sedentary lifestyles and are involved in lower levels of moderate to vigorous physical activity, which could lead to a poor CPF [33]. In previous studies that have evaluated the CPF of children and adolescents with VSDs using CPET, the results have been inconsistent [15,20,21,22,23]. The normalized peak VO_2_ ranged from 34 to 45 mL/min/kg, which is approximately 20–25% higher compared with our data (27.98–35.48 mL/min/kg) [15,20,21,23]. This difference might be related to ethnicity or different lifestyles. In previous studies, authors have analyzed the possible determinants of physical fitness in patients with CHD, with the results showing that active motivators are viewed as an important modifiable determinant [34]. Other studies have identified low self-efficacy, covert fears, discomfort and physical fatigue as limiting factors in children with CHD [35]. A lack of education concerning proper exercise could also be an important limiting factor [36]. Due to such factors, patients may have less active lifestyles and an inferior CPF compared with the norm.

Reviewing studies about the CPF of VSD patients, most patients are recruited in adulthood for evaluation of their long-term outcomes, and little CPET data for patients with isolated VSDs aged younger than 18 exists. As far as the CPF of adults diagnosed with VSDs is concerned, most studies have revealed an inferior CPF in either field tests or laboratory tests compared with that in a normal control group [10,11]. Possible mechanisms include abnormal cardiac contractility or an abnormal ventilation response [6,7,8,9,10,23].

In our study, a negative association between BMI and CPF was noticed in this population of young isolated VSD patients. In the general population within the same age range, a negative association between BMI and CPF has also been noticed [27,37]. In the general population of Taiwan, children with a normal weight status had an 8.73% to 16.2% higher normalized peak VO_2_ compared to that in overweight children in one study [27]. In one systemic review article that included nine studies and 478 cases at ages between 12 and 18 years, lean adolescents had a 7.35% to 70.6% higher normalized peak VO_2_ compared to that in obese adolescents [38].

In addition, congenital heart disease (CHD) survivors with obesity have a higher risk of cardiac-related morbidity and mortality as compared to that in those with a normal weight status [39,40]. Due to this negative association, body weight control is also a major issue for patients with VSDs.

This study had the following limitations. Firstly, our research was conducted in a single medical center, so its results might not apply to all isolated VSD patients. Secondly, patients with severely impaired cardiac functions and patients who refused to undergo CPET were excluded. Thirdly, our research only analyzed CPET data and demographic data without consideration of cardiac imaging nor the application of a daily activity questionnaire for a more comprehensive analysis. Lastly, a national survey of the participation of the Taiwanese population of young VSD patients in physical activity is lacking, which would provide additional evidence to support our inference about the causes of the sex difference.

## 5. Conclusions

In conclusion, male patients with isolated VSDs had a similar or better CPF compared with female patients. BMI had a negative association with CPF in young patients with VSDs. Further investigations in the form of prospective and well-designed studies are needed to confirm the conclusions of our study.

## Figures and Tables

**Table 1:** Comparison of body composition between two sexes in relation to age group.

Age Group	Age 5–9			Age 10–13			Age 14–18			All Age		
**Sex**	Boys (*n* = 44)	Girls (*n* = 35)	*p*-value	Boys (*n* = 44)	Girls (*n* = 50)	*p*-value	Boys (*n* = 69)	Girls (*n* = 47)	*p*-value	Boys (*n* = 157)	Girls (*n* = 132)	*p*-value
**Height**	126.16 ± 9.42	128.10 ± 8.79	0.350	148.72 ± 12.09	146.32 ± 13.44	0.672	168.19 ± 8.97	157.05 ± 5.88	<0.001	150.95 ± 20.1	145.31 ± 15.12	0.007
**Weight**	28.85 ± 9.72	29.64 ± 7.75	0.695	43.31 ± 12.40	41.55 ± 10.17	0.945	65.02 ± 17.46	51.37 ± 8.20	<0.001	48.8 ± 20.9	41.88 ± 12.26	0.001
**BMI**	17.71 ± 3.52	17.88 ± 3.39	0.833	19.21 ± 4.17	19.66 ± 4.20	0.289	22.91 ± 5.60	20.61 ± 3.04	0.005	20.42 ± 5.2	19.52 ± 3.74	0.092

BMI, body mass index; *p*-values: comparisons between boys and girls.

**Table 2:** Comparison of cardiopulmonary fitness during exercise testing between two sexes in relation to age group.

Age Group		ATVO_2_	ATVO_2_	PEAKVO_2_	PEAKVO_2_		Peak RER
		(mL/kg/min)	(mL/min)	(mL/kg/min)	(mL/min)		(N/A)
Ages 5–9	Boys (*n* = 44)	25.42 ± 5.37	722.99 ± 262.23	35.48 ± 6.43	1007.08 ± 354.64		1.14 ± 0.09
	Girls (n = 35)	25.38 ± 4.56	736.31 ± 170.27	35.17 ± 3.87	1029.05 ± 228.95		1.16 ± 0.08
	*p*-value	0.970	0.796	0.794	0.752		0.343
Ages 10–13	Boys (*n* = 44)	23.71 ± 3.87	1001.16 ± 224.52	35.13 ± 5.91	1477.80 ± 316.98		1.15 ± 0.08
	Girls (*n* = 50)	21.74 ± 4.41	894.86 ± 246.78	30.28 ± 5.46	1243.56 ± 318.25		1.16 ± 0.10
	*p*-value	0.024	0.032	<0.001	0.001		0.569
Ages 14–18	Boys (*n* = 69)	22.05 ± 4.80	1402.38 ± 367.46	32.93 ± 8.00	2086.66 ± 549.93		1.18 ± 0.08
	Girls (*n* = 47)	20.01 ± 4.58	1024.22 ± 267.92	27.98 ± 6.24	1423.17 ± 328.84		1.22 ± 0.12
	*p*-value	0.024	<0.001	0.001	<0.001		0.063
Age 5–18	Boys (*n* = 157)	23.46 ± 4.91	1099.54 ± 418.32	34.26 ± 7.10	1613.47 ± 634.14		1.16 ± 0.09
	Girls (*n* = 132)	22.09 ± 4.95	898.88 ± 261.18	30.76 ± 6.07	1250.63 ± 336.57		1.18 ± 0.10
	*p*-value	0.019	<0.001	<0.001	<0.001		0.071
**Age Group**		**Rest SBP**	**Rest DBP**	**Rest HR**	**Peak HR**	**Peak SBP**	**Peak DBP**
		**(mmHg)**	**(mmHg)**	**(beat/min)**	**(beat/min)**	**(mmHg)**	**(mmHg)**
Ages 5–9	Boys (*n* = 44)	101.61 ± 11.17	61.41 ± 7.92	88.73 ± 16.20	175.09 ± 15.27	146.0 ± 24.1	81.2 ± 17.3
	Girls (*n* = 35)	99.89 ± 12.74	60.69 ± 9.02	89.77 ± 14.51	179.09 ± 8.50	155.6 ± 35.4	80.4 ± 19.5
	*p*-value	0.523	0.706	0.767	0.145	0.175	0.850
Ages 10–13	Boys (*n* = 44)	114.09 ± 14.73	67.30 ± 7.80	87.11 ± 14.86	176.09 ± 9.27	159.9 ± 24.1	83.8 ± 21.3
	Girls (*n* = 50)	107.96 ± 13.68	63.84 ± 8.17	86.86 ± 11.70	175.90 ± 14.65	156.0 ± 29.8	85.7 ± 20.2
	*p*-value	0.039	0.039	0.927	0.941	0.487	0.655
Ages 14–18	Boys (*n* = 69)	122.97 ± 17.15	71.29 ± 10.80	83.68 ± 13.02	177.70 ± 14.88	179.5 ± 27.6	91.5 ± 21.5
	Girls (*n* = 47)	116.04 ± 18.54	69.81 ± 9.25	82.94 ± 10.34	172.28 ± 15.40	163.7 ± 30.3	90.2 ± 21.7
	*p*-value	0.041	0.444	0.743	0.060	0.005	0.751
Ages 5–18	Boys (*n* = 157)	114.50 ± 17.35	67.40 ± 10.09	86.06 ± 14.56	176.52 ± 13.62	164.7 ± 29.2	86.5 ± 20.8
	Girls (*n* = 132)	108.70 ± 16.52	65.13 ± 9.48	86.23 ± 12.28	175.45 ± 13.77	158.6 ± 31.5	85.9 ± 20.8
	*p*-value	0.04	0.051	0.912	0.512	0.90	0.804

AT VO_2_, oxygen consumption at anaerobic threshold; N/A, non-applicable; peak VO_2_, peak oxygen consumption during exercise testing; RER, respiratory exchange ratio. *p*-values: comparisons between boys and girls. SBP, systolic blood pressure; DBP, diastolic blood pressure; HR, heart rate.

**Table 3:** Correlation between body mass index and performance in the exercise test of children and adolescents.

Age Group			ATVO_2_	ATVO_2_	PEAKVO_2_	PEAKVO_2_
			(mL/kg/min)	(mL/min)	(mL/kg/min)	(mL/min)
Age 5–9	Boys (*n* = 44)	BMI	−0.306 *	0.664 **	−0.386 **	0.664 **
	Girls (*n* = 35)	BMI	−0.527 **	0.452 **	−0.557 **	0.636 **
	Total (*n* = 79)	BMI	−0.392 **	0.584 **	−0.430 **	0.644 **
Age 10–13	Boys (*n* = 44)	BMI	−0.580 **	0.539 **	−0.599 **	0.536 **
	Girls (*n* = 50)	BMI	−0.297 *	0.164	−0.347 *	0.151
	Total (*n* = 94)	BMI	−0.420 **	0.309 **	−0.452 **	0.290 **
Age 14–18	Boys (*n* = 69)	BMI	−0.459 **	0.473 **	−0.506 **	0.382 **
	Girls (*n* = 47)	BMI	−0.100	0.412 **	−0.329 *	0.215
	Total (*n* = 116)	BMI	−0.288 **	0.502 **	−0.351 **	0.415 **
Age 5–18	Total (*n* = 289)	BMI	−0.425 **	0.568 **	−0.447 **	0.532 **

* *p*-value < 0.05, ** *p*-value < 0.01. AT VO_2_, oxygen consumption at anaerobic threshold; BMI, body mass index; peak VO_2_, peak oxygen consumption during exercise testing. Data are presented as Pearson’s coefficient factors (*p*-values).

## Data Availability

The original contributions presented in this study are included in the article. Further inquiries can be directed to the corresponding author(s).
